# Analysis of Unique Flavor Metabolites and Seasonal Variations of the Special Tea Plant Cultivar of Fuliang Tea, a Geographical Indication Product

**DOI:** 10.3390/plants15111635

**Published:** 2026-05-26

**Authors:** Cuinan Yue, Xinmin Wang, Chunyun Zhang, Kebiao Chen, Aizi Yuan, Bin Zhang, Hao Xu, Puxiang Yang, Wenjin Li, Yongxin Wang, Zhihui Wang

**Affiliations:** 1 Jiangxi Provincial Key Laboratory of Plantation and High Valued Utilization of Specialty Fruit Tree and Tea, Jiangxi Cash Crops Research Institute, Nanchang 330043, China; m18306083532@163.com (C.Y.);; 2 College of Agriculture, Jiangxi Agricultural University, Nanchang 330045, China; 3 Jing’an County Agriculture and Rural Bureau, Yichun 330699, China; 4 Fuliang County Tea Industry Development Center, Jingdezhen 333499, China; 5 Key Laboratory of Tea Biology and Resources Utilization, Ministry of Agriculture, National Center for Tea Improvement, Tea Research Institute, Chinese Academy of Agricultural Sciences, Hangzhou 310008, China

**Keywords:** tea plant, cultivar, green tea, Fuliang tea, flavor, flavoromics, metabolites

## Abstract

This study investigated the key flavor metabolites and their seasonal variation mechanisms in the Fuliang green tea-specific cultivar ‘Fuliang Zhuye 1’ (FLZY). Results showed that cultivar identity shaped the metabolite profile more strongly than spring seasonal changes. Compared with ‘Fuding Dabaicha’ (FDDB), FLZY better represented the traditional flavor profile of Fuliang green tea, which is characterized by floral aroma and a sweet aftertaste. Using multimodal flavoromics, we identified a set of key aroma- and taste-active compounds capable of reconstituting FLZY’s characteristic flavor. Omission experiments suggested that several amino acids—including L-theanine, L-glutamic acid, L-aspartic acid, and L-glutamine—enhanced umami, sweetness, mellowness, and sweet aftertaste. These amino acids also reduced bitterness and astringency. Notably, their individual dose-over-threshold values were all below one. The springtime decline of these components correlated with a reduction in overall flavor intensity. Furthermore, FLZY accumulated markedly higher levels of kaempferol-3-*O*-rutinoside than FDDB. This difference may have contributed to its enhanced sweet aftertaste. These findings provide references for protecting the traditional flavor of geographical indication tea products and for targeted tea plant breeding.

## 1. Introduction

Geographical indication agricultural products derive their unique flavor qualities from local cultivars, natural environments, and human factors [1]. Fuliang green tea (hereafter “Fuliang tea”) is a geographical indication product from Fuliang County, Jiangxi Province, China. With a history of over 2000 years, it is exported to more than 60 countries and regions worldwide [2]. Fuliang tea is known for a tight, straight appearance, an orchid-like aroma, a mellow and brisk taste, and a sweet aftertaste [3]. The traditional Fuliang population cultivar plays an important role in forming these unique sensory characteristics. However, this cultivar has limitations, including late spring harvest and low yield, which severely impact the economic benefits of Fuliang tea [4]. Over the past 40 years, tea enterprises in Fuliang County have introduced clonal-improved cultivars like ‘Fuding Dabaicha’. Nevertheless, tea produced from these cultivars lacks distinction, leading to a loss of the unique quality traits traditional to Fuliang tea [4]. To address this, our team spent over a decade improving the Fuliang population cultivar, ultimately selecting and breeding the high-quality, clonal cultivar ‘Fuliang Zhuye 1’, which features early germination and high yield. Tea made from ‘Fuliang Zhuye 1’ retains the traditional quality characteristics of Fuliang tea and has been widely promoted [4]. However, a critical knowledge gap remains: although both cultivar-specific traits and seasonal variations are known to influence tea flavor [5,6], their relative contributions to the unique sensory profile and flavor metabolites of Fuliang tea have not been systematically compared.

Tea contains abundant flavor metabolites, including non-volatile compounds like catechins, amino acids, flavonol glycosides, nucleotides, and organic acids, as well as volatile terpenes, alcohols, aldehydes, esters, and heterocycles [7,8]. Variations in the composition and proportion of these metabolites contribute to diverse tea flavors [9]. Cultivars can specifically accumulate characteristic metabolites, conferring cultivar specificity [10]. Recent advances in metabolomics and flavoromics have enabled the systematic dissection of cultivar-specific flavor signatures [11,12]. For instance, ‘Jianghua Kucha’ exhibits intense bitterness due to the specific accumulation of 1,3,7-trimethyluric acid, methylated epigallocatechin gallate (EGCG), and purine alkaloids [13]. Similarly, the oolong tea cultivar ‘Huangmeigui’ accumulates phenylacetaldehyde and 3,5-diethyl-2-methylpyrazine, giving it a unique aroma [14]. Empirical production experience suggests that ‘Fuliang Zhuye 1’ yields tea with a stronger floral aroma and sweeter aftertaste compared to other cultivars. From a flavoromics perspective, ‘Fuliang Zhuye 1’ is mechanistically interesting. Its genetic background is derived from the traditional Fuliang population cultivar [4]. This background may enable the coordinated accumulation of specific volatile and non-volatile metabolites via key biosynthetic pathways (e.g., phenylalanine metabolism, terpenoid biosynthesis) [15,16]. Such accumulation could potentially explain its superior sensory traits. This contrasts with ‘Fuding Dabaicha’, which is a widely introduced cultivar [17]. ‘Fuding Dabaicha’ does not produce the same characteristic flavor [4]. Therefore, this cultivar pair serves as an ideal model for dissecting the metabolic basis of geographical indication product authenticity. Previous studies indicate that the main contributors to floral aroma in green tea include geraniol, linalool, nerol, phenylethanol, and ionones [18,19]. Significant contributors to the sweet aftertaste of tea infusions primarily include tea polyphenols (especially their hydrolysis products), amino acids, soluble sugars, and organic acids [20,21]. We hypothesize that these components may be key to the pronounced floral aroma and sweet aftertaste of ‘Fuliang Zhuye 1’.

Tea flavor is also influenced by seasonal variation [22]. For green tea, the spring season is vitally important, with spring tea output accounting for over 70% of the annual total value [23]. Spring tea is further categorized into early, mid, and late spring [24]. Studies have demonstrated that seasonal metabolic shifts, particularly in nitrogen and carbon assimilation, alter the balance of amino acids, catechins, and flavonol glycosides, thereby modulating tea quality [25]. Additionally, 53 differential metabolites, including catechins, amino acids, and flavonoid glycosides, have been identified as contributors to flavor variations in tea across different spring periods [26]. In early spring, nitrogen metabolism in tea plants is vigorous, while carbon metabolism intensifies in late spring. Consequently, amino acid content gradually decreases while tea polyphenol content increases, weakening the fresh, brisk taste and intensifying the bitterness and astringency of the tea infusion [19,27]. Therefore, seasonal changes during spring may also affect the quality of Fuliang tea produced from ‘Fuliang Zhuye 1’ by altering metabolite content. Despite the agronomic and sensory importance of spring harvest timing, the interplay between cultivar-dependent flavor potential and seasonally driven metabolic fluctuations remains poorly understood for premium Fuliang tea cultivars.

Based on this, using ‘Fuding Dabaicha’ as a control, this study employed a multimodal flavoromics approach to investigate the metabolic basis of the unique flavor formation in ‘Fuliang Zhuye 1’ and the influence of spring seasonal changes. The methods included quantitative descriptive analysis (QDA), volatile and non-volatile metabolomics, gas chromatography-olfactometry (GC-O), chemometrics, relative odor activity value (rOAV), dose-over-threshold (Dot) analysis, flavor recombination, and omission experiments. The goal was to identify the small-molecule metabolites associated with the unique flavor of ‘Fuliang Zhuye 1’ and to explore how spring seasonal variation affects these flavor substances. These findings may serve as a reference for the targeted breeding of new tea plant cultivars for geographical indication products such as Fuliang tea and for identifying biochemical regulation nodes for the standardized production of spring tea.

## 2. Materials and Methods

### 2.1. Chemicals

Standards of L-theanine, L-aspartic acid, L-histidine, L-glutamic acid, *β*-alanine, L-tyrosine, L-proline, serine, L-lysine, L-tryptophan, L-leucine, L-arginine, L-glutamine, and phenylalanine (≥98%) were purchased from Waters (Milford, MA, USA). Standards of citric acid, glucose, malic acid, and fructose (≥98%) were purchased from Yuanye (Shanghai, China). Standards of methyl salicylate, linalool, indole, heptanal, geraniol, decanal, benzeneacetaldehyde, 3-ethyl-2,5-dimethyl-pyrazine, (Z)-3-hexen-1-ol, nonanal, D-limonene, caffeine, quercetin, quercetin-3-*O*-glucoside, gallic acid (GA), kaempferol-3-*O*-galactoside, gallocatechin gallate (GCG), quercetin-3-*O*-galactoside, epigallocatechin gallate (ECG), kaempferol, epicatechin (EC), catechin (C), myricetin, myricetin-3-*O*-rutinoside, quercetin-3-*O*-rutinoside, EGCG, myricetin-3-*O*-galactoside, kaempferol-3-*O*-glucoside, epigallocatechin (EGC), myricetin-3-*O*-glucoside, kaempferol-3-*O*-rutinoside, and normal alkanes (C7-C40) (≥98%) were purchased from Aifa (Chengdu, China).

### 2.2. Tea Samples

**Figure 1:**
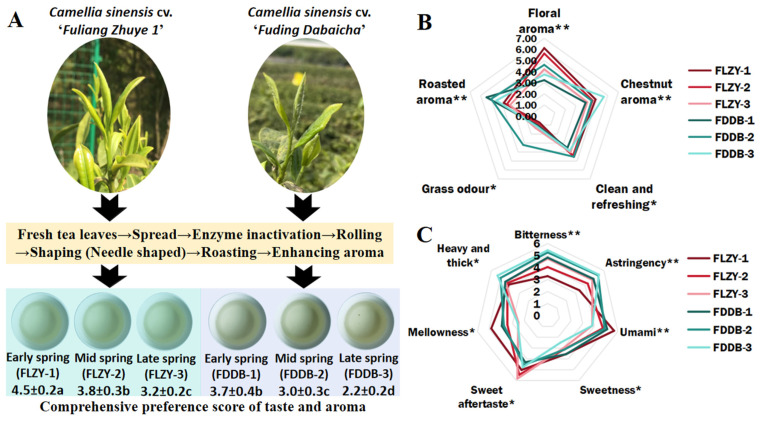
Comparison of sensory characteristics between ‘Fuliang Zhuye 1’ (FLZY) and ‘Fuding Dabaicha 1’ (FDDB). (**A**) The production of each sample and the evaluation of preferences. Different letters indicate significant differences at the *p* < 0.05 level. (**B**) Aroma intensity profiles of FLZY and FDDB. (**C**) Taste intensity profiles of FLZY and FDDB. * *p* < 0.05, ** *p* < 0.01.

Both ‘Fuding Dabaicha’ and ‘Fuliang Zhuye 1’ are clonal cultivars (Figure 1A) and were planted in the same plot in Jiangxi Province (28°22′20″ N, 116°0′6″ E) under identical management practices. In the spring of 2023, fresh leaves (one bud and two leaves) from both cultivars were harvested on 28 March (early spring, temperature 15–17 °C, relative humidity 68–72%), 5 April (mid-spring, temperature 17–19 °C, relative humidity 65–70%), and 20 April (late spring, temperature 25–28 °C, relative humidity 60–65%). All samples were processed into Fuliang tea uniformly following DB36/T 1062-2018 (Technical Regulations for Production of Fuliang Tea, a Jiangxi Provincial Local Standard) (Figure 1A). The processing procedure was as follows: fresh tea leaves → spreading (thickness: 1 cm, time: 3 h) → enzyme inactivation (rotary drum temperature: 220 °C, time: 3.5 min) → rolling (time: 20 min) → shaping (needle-shaped; tea straightening machine temperature: 100 °C, time: 20 min) → roasting (temperature: 90 °C, time: 10 min) → enhancing aroma (temperature: 80 °C, time: 20 min). The tea produced from ‘Fuliang Zhuye 1’ was designated FLZY. The FLZY samples from the three spring periods were named FLZY-1 (early spring), FLZY-2 (mid-spring), and FLZY-3 (late spring). The tea produced from ‘Fuding Dabaicha’ was designated FDDB, with corresponding samples named FDDB-1, FDDB-2, and FDDB-3. Three biological replicates were used for the preparation of each sample. All samples were stored at −80 °C until analysis. Voucher specimens of the two clonal cultivars were collected and deposited at the Herbarium of Jiangxi Cash Crops Research Institute (Nanchang, China) under accession numbers JXCCRI-TEA-FD1 (Fuding Dabaicha) and JXCCRI-TEA-FZ1 (Fuliang Zhuye 1).

### 2.3. QDA

Prior to formal evaluation, all panelists underwent a standardized training program following GB/T 16291.1-2012 [26]. The training program included the following components: (1) attribute definition and terminology alignment, where an initial list of 5 aroma and 8 taste descriptors was generated through consensus-based discussion (Table S1); (2) reference standard calibration using multiple concentrations of reference substances (e.g., caffeine at 0.05–0.8 mg/mL for bitterness and glutamic acid at 0.04–0.32 mg/mL for umami), with panelists required to achieve three consecutive correct identifications in blind tests for each attribute (Table S1); (3) intensity scale anchoring with blind testing, where panelists practiced scoring at least three concentration levels per attribute and maintained a coefficient of variation (CV) < 15%; and (4) practice evaluations with individual feedback, where panelists evaluated three commercial Fuliang tea samples and repeated the exercise until each achieved a mean deviation < 15% from the group mean across all attributes. All nine panelists passed the final validation test and proceeded to formal QDA. The panel consisted of nine members (5 females, 4 males, aged 24–59 years) from the tea innovation team of the Jiangxi Cash Crops Research Institute. All panelists are tea science researchers with over three years of green tea evaluation experience.

Tea infusions were prepared according to GB/T 23776-2018 (Methodology for Sensory Evaluation of Tea) [28]. Briefly, 3.0 g of tea sample was placed in a cylindrical cup, brewed with 150 mL of boiling water for 4 min, and then decanted. The panel evaluated the intensity of each taste and aroma attribute as well as overall preference. Attribute intensity was scored on a scale of 0–7 (0 = none, 7 = extremely strong). Overall preference for taste and aroma was rated on a scale from −5 (extremely dislike) to +5 (extremely like). Panelists conducted assessments independently without discussion. A 5 min break was enforced between samples. All evaluations were performed with three biological replicates.

To assess inter-rater reliability, the intraclass correlation coefficient (ICC) was calculated using a two-way random effects model for absolute agreement. The ICC for the nine panelists was 0.86 (95% CI: 0.74–0.93), indicating good agreement among panelists. Additionally, Cronbach’s *α* was 0.89, confirming high internal consistency of the sensory evaluations.

### 2.4. Volatile Metabolome Analysis

For extraction, 500 mg of ground sample was placed in a 20 mL headspace vial. Then, 2.0 mL of saturated sodium chloride solution and 20 µL of 3-hexanone-2,2,4,4-D4 internal standard solution (10 µg/mL) were added, and the vial was immediately sealed. The sample was equilibrated at 60 °C for 5 min with agitation. Subsequently, a DVB/CWR/PDMS headspace solid-phase microextraction (HS-SPME) Arrow fiber (Agilent, Santa Clara, CA, USA) attached to a manual holder was exposed to the vial headspace for 15 min at 60 °C. The fiber was then immediately inserted into the injector of an 8890-7000E gas chromatography-tandem mass spectrometry (GC-MS/MS) system (Agilent, Santa Clara, CA, USA) for thermal desorption at 250 °C for 5 min. The GC oven temperature program was as follows: 40 °C (held for 3.5 min), increased to 100 °C at 10 °C/min, then to 180 °C at 7 °C/min, and finally to 280 °C at 25 °C/min (held for 5 min). Separation was performed on a DB-5MS capillary column (Agilent, Santa Clara, CA, USA). A quality control (QC) sample was prepared by pooling aliquots from all sample extracts. Volatile metabolites were identified by matching against the NIST mass spectral library, a self-established Matware database, and by comparing calculated retention indices (RIs, determined using C7-C40 normal alkanes) with reference values. The content of volatile metabolites was calculated based on the relative response to the internal standard, following a published formula [28].

The rOAV was used to evaluate the contribution of volatile metabolites to aroma perception. Generally, a volatile metabolite with rOAV > 1 is considered an important contributor to the overall aroma [14]. It was calculated as rOAV = C/T, where C is the concentration and T is the aroma threshold value of the volatile metabolite [26].

### 2.5. GC-O

The same extraction method as described for GC-MS/MS was employed. Metabolite separation was performed on an SH-I-5Sil MS column (Shimadzu, Tokyo, Japan). Volatile metabolites were identified using a GC-2030 GC system (Shimadzu) coupled with a QP2020 NX mass spectrometer (Shimadzu). An ODE-2030 olfactometer (Shimadzu) connected to the GC-MS was used to characterize the aroma attributes of volatile metabolites. The GC-O experimental parameters followed those described by Yue et al. (2026) [28]. The QDA panel evaluated the aroma intensity of detected volatile metabolites on a scale of 0 to 7. Each sample was analyzed in three biological replicates.

### 2.6. Non-Volatile Metabolome Analysis

Non-volatile metabolome profiling was performed using ultra-performance liquid chromatography-tandem mass spectrometry (UPLC-MS/MS) (Shimadzu, Kyoto, Japan). Fifty milligrams of powdered sample were weighed and placed in a centrifuge tube. Then, 1200 µL of 70% methanol (containing 2-chlorophenylalanine as an internal standard) was added. The mixture was extracted for 3 h, with vortexing performed 6 times during this period (30 s each). Subsequently, the sample was centrifuged at 13,400× *g* for 3 min. The supernatant was filtered through a 0.22 µm microporous membrane. Chromatographic separation was achieved using an ACQUITY UPLC HSS T3 Column (Waters, Milford, MA, USA). Mass spectrometry analysis was conducted on a TripleTOF 6600+ system (SCIEX, Foster City, CA, USA). Detailed conditions for chromatography and mass spectrometry analysis, as well as methods for qualitative and quantitative data analysis, followed published literature [29].

### 2.7. Absolute Quantification of Taste-Related Metabolites

Caffeine, GA, and six catechins were quantified using a 2695 HPLC system (Waters) equipped with an X-select-T3 column (Waters). Chromatographic conditions followed a published method [26]. Fifteen free amino acids were analyzed using the same 2695 HPLC system with an AccQ·Tag column (Waters). Pre-column derivatization was performed using 6-Aminoquinoline-N-hydroxysuccinimide carbamate derivatives. Other analytical conditions followed Wang, Zheng, et al. (2024) [30]. Twelve flavonols were determined using a 1260 HPLC system (Agilent, Santa Clara, CA, USA) with a ZORBAX ODS column (Agilent), following published chromatographic conditions [26].

Total tea polyphenols were determined using the Folin–Ciocalteu colorimetric method [26]. Total amino acids were quantified via the ninhydrin colorimetric method [26]. Water extract content was determined by the weight reduction method [26]. Contents of glucose, fructose, malic acid, and citric acid were measured using commercial assay kits (Grace Biotechnology Co., Ltd., Suzhou, China) according to the manufacturer’s instructions. The contents of all these components were expressed as milligrams per gram of dry weight. All analyses were performed with three biological replicates.

The Dot value was used to evaluate the contribution of non-volatile metabolites to taste perception. Generally, a non-volatile metabolite with Dot >1 is considered an important contributor to the overall taste [7]. It was calculated as Dot = C_i_/T_i_, where C_i_ is the concentration and T_i_ is the taste threshold value of the metabolite. Aroma and taste thresholds for metabolites in water were obtained from published literature [7,8,31,32].

### 2.8. Flavor Recombination and Omission Experiments

The flavor recombination method followed Cui et al. (2021) [33]. Three recombination experiments were conducted: Group 1: Eleven key aroma metabolites and thirteen key taste metabolites were added to pure water at concentrations matching those in the FLZY-1 tea infusion. Group 2: The compounds from Group 1, plus ten important taste metabolites (0.1 < Dot < 1), were added to pure water at concentrations matching the FLZY-1 infusion. Group 3: The compounds from Group 1, plus four important taste metabolites (0.1 < Dot < 1), were added to pure water at concentrations matching the FLZY-1 infusion.

The control (FLZY-1’s original tea infusion) was prepared by steeping 3.0 g of tea in 150 mL of boiling water for 4 min. The infusion was then filtered and cooled to room temperature in a cold-water bath. Sensory evaluation was performed as described for QDA.

Omission experiments followed Yue et al. (2026) [28]. Incomplete recombinants, each lacking one of the 33 flavor metabolites, and the complete recombinant were randomly coded for three-alternative forced-choice tests. The results were used for difference analysis.

### 2.9. Data Statistics and Analysis

Principal component analysis (PCA), second-order orthogonal projections to latent structures (O2PLS), hierarchical cluster analysis (HCA), and partial least squares discriminant analysis (PLS-DA) were performed using SIMCA software (version 14.0). Heat maps were generated using HIPLOT. One-way analysis of variance (ANOVA) with least significant difference and correlation analysis were performed using SPSS software (version 19.0). To account for the high dimensionality of metabolomics data and the risk of false positives due to multiple hypothesis testing, the Benjamini–Hochberg false discovery rate (FDR) procedure was applied. An adjusted *p*-value (q-value) < 0.05 was considered statistically significant. The network diagram was generated using Cytoscape software (version 3.9.1).

## 3. Results and Discussion

### 3.1. The Taste and Aroma Sensory Characteristics of FLZY and FDDB

‘Fuding Dabaicha’ is the most widely planted national-level tea plant cultivar in China and is suitable for processing various types of green tea. As a publicly known and commonly used cultivar, it is extensively cultivated in the Fuliang tea production area [4]. Therefore, this study used ‘Fuding Dabaicha’ (FDDB) as a control to investigate the flavor and characteristic metabolites of ‘Fuliang Zhuye 1’ (FLZY). The comprehensive preference score for the taste and aroma of FLZY (mean 3.83) exceeded that of FDDB (mean 2.97) (*p* < 0.05) (Figure 1A), suggesting the superior quality of FLZY. From early to late spring, the comprehensive preference score for FLZY gradually decreased, a trend also observed for FDDB. This is consistent with previously reported findings for the geographical indication product “Longjing green tea,” where the quality of early spring tea is higher than that of mid-spring and late spring tea [24].

For FLZY, the intensity of the floral aroma was the highest (mean 5.32) and was greater than that of FDDB (mean 3.86) (*p* < 0.05) (Figure 1B; Table S2). A floral aroma is a typical characteristic of traditional high-quality Fuliang tea [3], suggesting that FLZY is suitable for producing such tea. In contrast, the typical aroma characteristic of FDDB was a roasted aroma, which showed the strongest intensity (mean 5.00) and exceeded that in FLZY (mean 3.52) (*p* < 0.05). The “clean and refreshing” attribute was more intense in FLZY than in FDDB, whereas the “chestnut-like” aroma was more intense in FDDB. Although FDDB exhibited a stronger “grassy” odor than FLZY, the absolute intensity of this odor was low. From early to late spring, the intensities of the floral, chestnut-like, clean and refreshing, and roasted aromas in FLZY gradually weakened, while the grassy odor intensified. Previous studies indicate that floral, clean and refreshing, and chestnut-like aromas contribute positively to the aroma quality of green tea, whereas a grassy odor has a negative effect [34]. These seasonal changes may contribute to a decline in the aroma quality of FLZY over the spring season.

The unique taste characteristic of FLZY was a strong “sweet aftertaste”, which had the highest intensity (mean 5.43) and was greater than that in FDDB (mean 4.55) (*p* < 0.05). Conversely, the intensities of “bitterness” (mean 4.02) and “astringency” (mean 4.14) were lowest in FLZY and were lower than those in FDDB (*p* < 0.05) (Figure 1C; Table S3). FLZY also showed slightly stronger “umami”, “sweetness”, and “mellowness” than FDDB, while FDDB scored higher for “heavy and thick” (*p* < 0.05). Mellowness and a sweet aftertaste are typical taste characteristics of traditional high-quality Fuliang tea [3]. In addition to its pronounced sweet aftertaste, FLZY also exhibited relatively strong umami and mellowness, suggesting an improvement in taste quality compared to traditional Fuliang tea. The influence of seasonal progression on the taste attributes was consistent for both cultivars: from early to late spring, the intensities of umami, sweetness, and mellowness gradually weakened, while the intensities of bitterness, astringency, sweet aftertaste, and heavy and thick notes gradually increased (*p* < 0.05).

Overall, FLZY can produce higher-quality Fuliang tea. Its unique flavor profile is characterized by an extremely strong floral aroma and sweet aftertaste, coupled with relatively weak bitterness and astringency. Spring seasonal changes reduce the quality of FLZY, primarily due to the gradual weakening of floral aroma, umami, sweetness, and mellowness, alongside the gradual increase in grassy odor, bitterness, and astringency.

### 3.2. The Metabolite Basis of the Unique Aroma of FLZY

**Figure 2:**
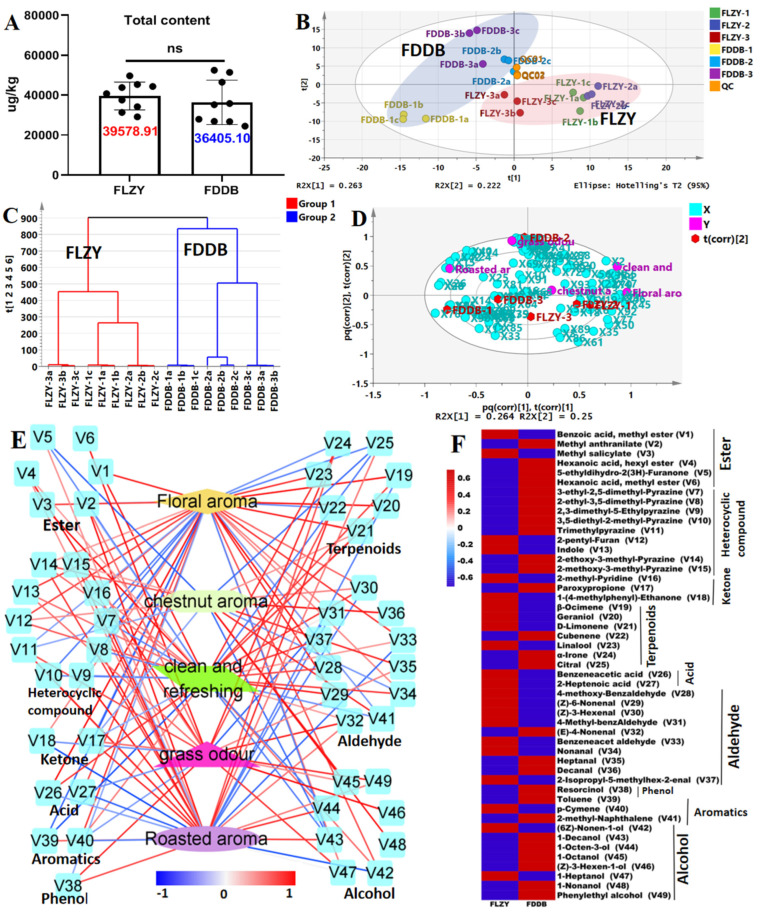
Comparison of aroma metabolites between ‘Fuliang Zhuye 1’ (FLZY) and ‘Fuding Dabaicha 1’ (FDDB). (**A**) Total volatile metabolite abundance in FLZY and FDDB. ns, no significance. (**B**) Principal component analysis distinguishing FLZY and FDDB based on volatile metabolites. R^2^X = 0.932; Q^2^ = 0.570. QC, quality control. (**C**) Clustering of volatile metabolites in FLZY and FDDB. (**D**) Second-order orthogonal projections to latent structures (O2PLS) loading plot identifying key volatile metabolites differentiating FLZY and FDDB. Seven-fold cross-validation was used. R^2^X = 0.855; R^2^Y = 0.936; Q^2^ = 0.794. The names of volatile metabolites X1–X95 are shown in Table S5. (**E**) Correlation network between key aroma metabolites and aroma attributes. V1–V49 are shown in (**F**). The red line indicates positive correlation; the blue line indicates negative correlation. (**F**) Heat map showing differential accumulation of key aroma metabolites between FLZY and FDDB. Red indicates high content and blue indicates low content. The content of each aroma metabolite of each cultivar is the average of its three samples.

A total of 223 volatile metabolites were identified, including 41 aldehydes, 37 terpenoids, 34 esters, 31 heterocyclic compounds, 29 alcohols, 17 ketones, 15 aromatics, 10 phenols, and 9 acids (Table S4). Although the total amount of volatile metabolites did not differ between FLZY and FDDB (*p* > 0.05), the content in FLZY (39,578.91 µg/kg) was higher than that in FDDB (36,405.10 µg/kg) (Figure 2A). This provides a quantitative basis for the better aroma quality of FLZY. In FLZY, terpenoids (mean 22.61%) and aldehydes (22.61%) accounted for a relatively high proportion of the total volatile metabolite content, whereas in FDDB, heterocyclic compounds (25.86%) constituted the highest proportion. Terpenoids and aldehydes are typically associated with prominent floral and fruity aromas, while heterocyclic compounds are generally linked to roasted aromas [9]. This may explain why FLZY is dominated by floral notes and FDDB by roasted notes.

PCA effectively separated FLZY and FDDB samples, indicating significant differences in their volatile metabolite profiles (Figure 2B). HCA also classified the two cultivars into distinct categories (Figure 2C), suggesting that the cultivar effect on volatile metabolites was greater than that of spring seasonal changes.

To explore the metabolic basis of FLZY’s unique aroma, a PLS-DA model was established (Figure S1). Based on the criteria of variable importance in projection (VIP) > 1 and q < 0.05 [35], 95 differential volatile metabolites were identified (Table S5). These metabolites may be associated with the distinct aroma of FLZY. To assess their contribution to specific aroma sub-attributes, correlation analysis and an O2PLS model were employed. The O2PLS loading plot showed that FLZY samples from all three periods clustered in the same quadrant as the “floral aroma” attribute, whereas FDDB samples were closer to the “roasted aroma” attribute (Figure 2D). This suggests that volatile metabolites in FLZY may contribute to its floral aroma, while those in FDDB may contribute to its roasted aroma, consistent with the sensory analysis results. Using O2PLS-VIP > 1, correlation coefficient |r| ≥ 0.7 and *p* < 0.05 as screening criteria [30], 49 volatile metabolites were identified as contributors to the five aroma sub-attributes. Figure 2E and Table S6 present the correlations between these 49 key aroma metabolites and the aroma attributes.

Compounds that contributed positively to the floral aroma included geraniol, linalool, phenylacetaldehyde, indole, 2-pentyl-furan, *β*-ocimene, phenylacetic acid, 4-methoxy-benzaldehyde, and D-limonene, all of which are known for their floral notes [32] (Table S4). The contents of these nine components were higher in FLZY than in FDDB (q < 0.05) (Figure 2F). Specifically, geraniol and phenylacetic acid contents in FLZY were 4.52 and 3.19 times those in FDDB, respectively (Table S6). Although the key floral compounds vary among green tea types, geraniol and linalool are often core contributors [36]. In FLZY, the synergistic effect of these two core compounds with the other seven components may contribute to its distinctive floral fragrance. The accumulated terpenoid floral compounds (geraniol, linalool, *β*-ocimene, D-limonene) in FLZY are primarily synthesized from precursors such as geranyl pyrophosphate catalyzed by terpene synthases [28]. They are often stored in non-volatile glycoside-bound forms (e.g., β-primeverosides) and hydrolyzed by *β*-primeverosidase during processing to release free volatiles [18]. The phenylpropanoid/benzenoid compounds (benzeneacetaldehyde, benzeneacetic acid, 4-methoxybenzaldehyde) are derived from phenylalanine via phenylalanine ammonia-lyase and subsequent enzymatic reactions [19]. Indole is produced through the tryptophan metabolic pathway, while 2-pentyl-furan originates from the lipoxygenase pathway [28]. These pathways may act synergistically to contribute to the unique floral aroma of FLZY.

Fifteen compounds contributed positively to the roasted aroma, primarily including 2-ethoxy-3-methyl-pyrazine, 2-methoxy-3-methyl-pyrazine, 3-ethyl-2,5-dimethyl-pyrazine, 1-octen-3-ol, citral, 2-ethyl-3,5-dimethyl-pyrazine, 5-ethyldihydro-2(3H)-furanone, and 1-decanol. The contents of these components were all higher in FDDB than in FLZY (q < 0.05) (Figure 2F). Pyrazines and furans are known for their roasted aroma characteristics [37], while 1-octen-3-ol and 1-decanol impart fatty notes (Table S4). Studies suggest that fatty notes can directly enhance the intensity of a roasted aroma [32].

The primary compounds that contributed positively to the chestnut-like aroma were 1-octanol, heptanal, 2-methyl-naphthalene, decanal, and 1-nonanol, all of which were less abundant in FLZY than in FDDB (q < 0.05). Thirteen compounds contributed to the “clean and refreshing” attribute, mainly (Z)-3-hexenal, nonanal, methyl salicylate, linalool, and D-limonene, with higher contents in FLZY. Nine compounds contributed positively to the grassy odor, primarily 1-heptanol, (Z)-3-hexen-1-ol, (E)-4-nonenal, α-irone, and phenylethyl alcohol. Except for 1-heptanol, the contents of these components were higher in FDDB than in FLZY. Previous research has identified 1-heptanol and (Z)-3-hexen-1-ol as major sources of grassy odor in green tea [38], while the other three components may influence this attribute through interactive effects.

In summary, tea plant cultivar differences had a greater impact on the volatile metabolites of Fuliang tea than seasonal changes. Forty-nine important aroma metabolites were associated with the formation of FLZY’s unique aroma profile. The high accumulation of the nine metabolites that are positively associated with floral aroma in FLZY may contribute to the intense floral character of this tea. Further analysis, such as rOAV and GC-O, was conducted to screen for key aroma components.

### 3.3. Identification of Key Aroma Metabolites in FLZY

Using GC-O, 26, 28, and 27 volatile metabolites were detected in FLZY-1, FLZY-2, and FLZY-3, respectively, with diverse aroma characteristics such as floral, fruity, sweet, roasted, and green notes. In tea, a volatile metabolite with a rOAV greater than 1 is considered a key aroma component, as its aroma is perceivable [32]. By querying the aroma thresholds of each volatile metabolite in water, their rOAVs were calculated. Across the three FLZY samples, 11 volatile metabolites had an rOAV > 1. All 11 were among the aroma metabolites detected by GC-O and were also included in the 49 important aroma metabolites screened by O2PLS, suggesting that they may be key aroma components of FLZY.

**Figure 3:**
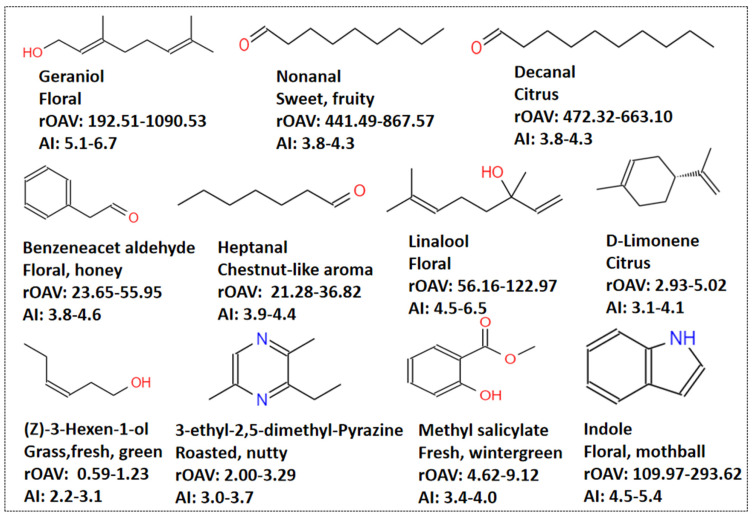
Key aroma components in ‘Fuliang Zhuye 1’ (FLZY) identified by relative odor activity value (rOAV) and gas chromatography-olfactometry (GC-O) analysis, with their aroma characteristics and intensities.

Figure 3 presents the aroma intensity (AI), GC-O-perceived aroma characteristics, and rOAV of these 11 components. In the O2PLS model, geraniol, linalool, indole, benzeneacetaldehyde, and D-limonene contributed positively to floral aroma. Except for D-limonene, the aroma characteristics of the other four components detected by GC-O were floral. Among them, geraniol, linalool, and indole exhibited the strongest AI values (greater than 4.5). Furthermore, geraniol (mean content: 4864.02 µg/kg) and indole (mean content: 8600.79 µg/kg) were the most abundant aroma components in FLZY (Table S4). The aroma characteristic of D-limonene detected by GC-O was citrus-like; this component may interact with floral compounds to enhance the overall floral fragrance. These five components may form the core skeleton of FLZY’s floral aroma. In other green teas, geraniol is typically found in higher content, while indole content is relatively lower [33]. However, in FLZY, the indole content was 1.77 times that of geraniol. Indole is recognized as a characteristic floral component in Oolong tea, where it is often present in relatively high concentrations [39]. Thus, the high indole content in FLZY may impart a distinctive floral character.

In the GC-O analysis, heptanal exhibited a chestnut-like aroma, methyl salicylate was fresh and wintergreen-like, (Z)-3-hexen-1-ol was grassy, and 3-ethyl-2,5-dimethyl-pyrazine was roasted. According to O2PLS, decanal and heptanal contributed to chestnut aroma; nonanal and methyl salicylate contributed to clean and refreshing notes; (Z)-3-hexen-1-ol contributed to grassy odor; and 3-ethyl-2,5-dimethyl-pyrazine contributed to roasted aroma. These components may constitute the core skeleton for the chestnut, clean and refreshing, grassy, and roasted aroma notes in FLZY. In conclusion, these 11 key aroma components may play a dominant role in the formation of FLZY’s unique aroma profile.

### 3.4. The Metabolite Basis of the Unique Taste of FLZY

**Figure 4:**
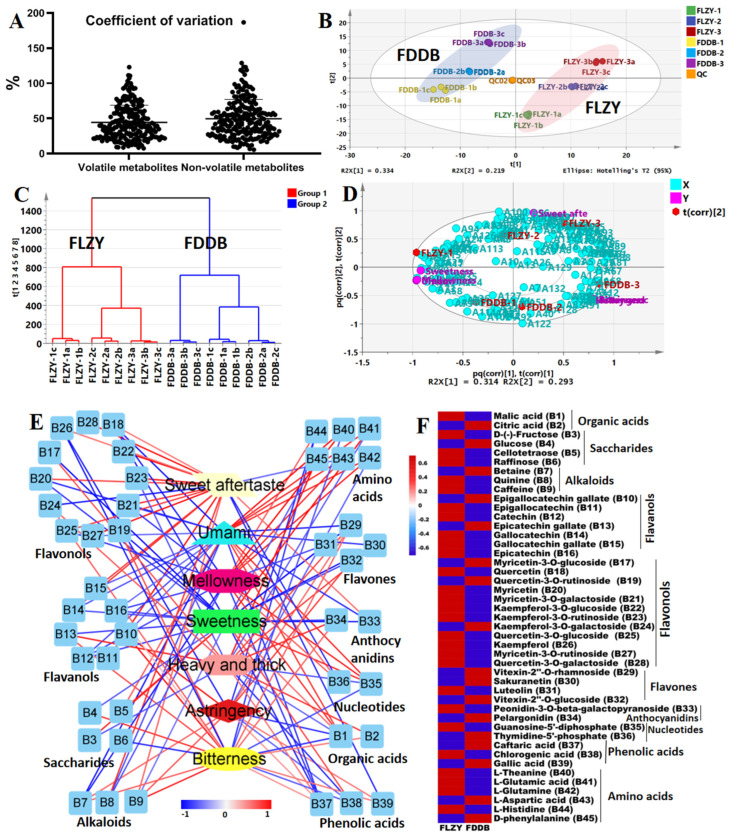
Comparison of non-volatile metabolites between ‘Fuliang Zhuye 1’ (FLZY) and ‘Fuding Dabaicha 1’ (FDDB). (**A**) Comparison of coefficient of variation between non-volatile and volatile metabolites. (**B**) Principal component analysis distinguishing FLZY and FDDB based on non-volatile metabolites. R^2^X = 0.825; Q^2^ = 0.618. QC, quality control. (**C**) Clustering of non-volatile metabolites in FLZY and FDDB. (**D**) Second-order orthogonal projections to latent structures (O2PLS) loading plot identifying key non-volatile metabolites differentiating FLZY and FDDB. Seven-fold cross-validation was used. R^2^X = 0.999; R^2^Y = 0.999; Q^2^ = 0.995. The names of non-volatile metabolites X1–X116 are shown in Table S5. (**E**) Correlation network between key taste metabolites and taste attributes. The red line indicates positive correlation; the blue line indicates negative correlation. B1–B45 are shown in Figure 4F. (**F**) Heat map showing differential accumulation of key taste metabolites between FLZY and FDDB. Red indicates high content and blue indicates low content. The content of each taste metabolite of each cultivar is the average of its three samples.

A total of 279 non-volatile metabolites were identified (Table S9), categorized as 85 flavonoids, 48 nucleotides, 35 organic acids, 22 amino acids, 22 phenolic acids, 19 saccharides, 19 saponins, 10 tannins, 9 alkaloids, 7 lipids, and 3 vitamins. Based on the coefficient of variation, the influence of cultivar differences on non-volatile metabolites (average CV: 44.40%) was greater than on volatile metabolites (average CV: 40.56%) in FLZY (Figure 4A), suggesting a greater potential for improving non-volatile metabolites in tea plants.

The results of PCA and HCA were consistent, clearly distinguishing the samples of the two cultivars into two separate groups (Figure 4B,C). This indicates that the non-volatile metabolites of the two cultivars differ substantially and that the differences in non-volatile metabolites driven by the tea plant cultivar itself have a greater impact than the seasonal variation in spring. This finding is similar to the results of the volatile metabolite analysis described above (Figure 2B,C). Previous studies have shown that differences in flavor metabolites due to cultivars cannot be fully compensated by cultivation or processing techniques. For example, L-theanine accumulation is more influenced by cultivar than by other factors [40], which is consistent with the results of this study.

To explore the metabolic basis of FLZY’s unique taste, a PLS-DA model was established (Figure S2). Based on the criteria of VIP > 1 and q < 0.05 [35], 134 differential non-volatile metabolites were screened (Table S10), which may contribute to FLZY’s distinctive taste. To investigate their specific contributions to taste sub-attributes, correlation analysis and an O2PLS model were performed. The O2PLS loading plot showed that the three FLZY samples were closely associated with sweet aftertaste, umami, sweetness, and mellowness, whereas the three FDDB samples were closer to bitterness, astringency, and heavy and thick mouthfeel (Figure 4D). This result is consistent with the aforementioned taste sensory analysis. Using O2PLS-VIP > 1, correlation coefficient |r| ≥ 0.7 and *p* < 0.05 as screening criteria [30], 45 metabolites were identified as contributors to the seven taste sub-attributes. Figure 4E and Table S11 show the correlations between these 45 important taste metabolites and the seven taste sub-attributes.

Flavonol glycosides and catechins, including kaempferol-3-*O*-glucoside, myricetin-3-*O*-rutinoside, EGC, quercetin-3-*O*-galactoside, kaempferol-3-*O*-rutinoside, quinine, myricetin-3-*O*-galactoside, GC, GCG, peonidin-3-*O*-beta-galactopyranoside, quercetin-3-*O*-glucoside, myricetin, and kaempferol, contributed positively to a sweet aftertaste. Their reported taste characteristics are velvety-like astringency, bitterness, and astringency [30]. Sweet aftertaste is not directly induced by sugars but is a delayed sweet sensation resulting from the combined effects of diminishing bitterness/astringency, the release of potential sweet compounds, and taste contrast in the oral cavity. It is a highly important and pleasant characteristic of high-quality green tea [21]. Some studies also indicate that flavonol glycosides can be hydrolyzed in the mouth to release glycosyl groups, which then interact with catechins, amino acids, and organic acids to produce a sweet aftertaste [8]. The contents of these 13 components were higher in FLZY than in FDDB, which may be a key reason for FLZY’s pronounced sweet aftertaste.

The main compounds contributing to umami were L-theanine, L-glutamine, guanosine-5′-diphosphate, L-glutamic acid, L-aspartic acid, and thymidine-5′-phosphate, whose taste characteristics are primarily umami and sweet [30]. Except for L-aspartic acid and thymidine-5′-phosphate, the contents of the other four were higher in FLZY than in FDDB (q < 0.05). Key contributors to sweetness included L-glutamic acid, guanosine-5′-diphosphate, L-theanine, cellotetraose, chlorogenic acid, and D-(*−*)-fructose, all of which were more abundant in FLZY. Compounds contributing to mellowness were L-theanine, guanosine-5′-diphosphate, L-glutamine, L-aspartic acid, malic acid, raffinose, and L-histidine (q < 0.05). Except for L-aspartic acid, the contents of the other six were higher in FLZY. Major contributors to bitterness were EGCG, caffeine, betaine, vitexin-2′-*O*-rhamnoside, and GA, which are characterized mainly by bitterness and astringency [8]. All five were more abundant in FDDB. Key contributors to astringency were EGCG, kaempferol-3-*O*-galactoside, myricetin-3-*O*-glucoside, catechin, and EC, also associated with bitterness and astringency [8]. Except for catechin, the other four were higher in FDDB. Components contributing to heavy and thick included glucose, D-phenylalanine, vitexin-2”-*O*-glucoside, ECG, quercetin-3-*O*-rutinoside, pelargonidin, EGCG, citric acid, sakuranetin, quercetin, caftaric acid, and luteolin. Except for quercetin and luteolin, the other 10 were more abundant in FDDB (q < 0.05).

In summary, tea plant cultivar differences had a substantial impact on the non-volatile metabolite profiles of Fuliang tea. Forty-five important taste metabolites were associated with the formation of FLZY’s unique taste. Thirteen taste components (mainly flavonols and their glycosides) with positive contributions to sweet aftertaste were highly accumulated in FLZY, potentially explaining its strong sweet aftertaste. Subsequently, absolute quantification and Dot analysis of taste metabolites were performed to screen for key taste components.

### 3.5. Identification of Key Taste Metabolites in FLZY

Absolute quantitative analysis was performed on forty-eight non-volatile metabolites (Table S12). Macroscopically, the average contents of tea polyphenols and total catechins in FLZY were 199.80 mg/g and 117.92 mg/g, respectively, both lower than those in FDDB. The average total free amino acid content in FLZY was 40.25 mg/g, higher than that in FDDB (38.72 mg/g). The average total flavonol glycoside content in FLZY was 4.89 mg/g, which was 2.08 times higher than that in FDDB (2.35 mg/g). High levels of tea polyphenols and catechins impart bitterness and astringency to tea infusions, whereas high free amino acid content contributes to umami and briskness [7]. The relatively higher free amino acid content combined with lower tea polyphenol and catechin levels in FLZY may contribute to its superior taste quality. The absolute metabolite concentrations were consistent with the trends observed in the aforementioned metabolomics data.

The concentrations of kaempferol-3-*O*-rutinoside, quercetin-3-*O*-galactoside, myricetin-3-*O*-rutinoside, and kaempferol-3-*O*-glucoside in FLZY were 11.75, 3.73, 2.33, and 2.24 times higher than those in FDDB, respectively. Kaempferol-3-*O*-rutinoside was the most abundant flavonol glycoside in FLZY (average 2.35 mg/g), compared to only 0.20 mg/g in FDDB. Its content in Longjing green tea is approximately 0.05 mg/g [41]. Therefore, FLZY may be a cultivar that specifically accumulates kaempferol-3-*O*-rutinoside. Consistent with previous analyses, this compound enhanced the sweet aftertaste, potentially explaining the notably strong sweet aftertaste of FLZY.

**Figure 5:**
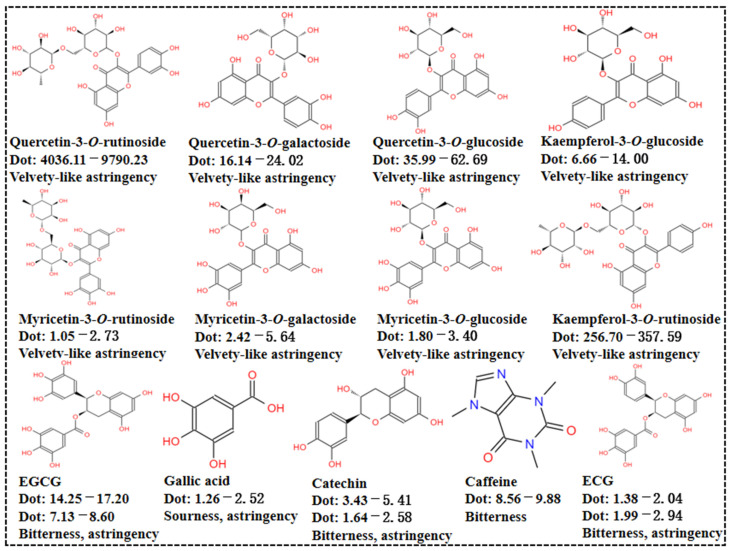
Key taste metabolites in ‘Fuliang Zhuye 1’ (FLZY) with their chemical structures, taste characteristics, and dose-over-threshold (Dot) values. ECG, epigallocatechin gallate; EGCG, epigallocatechin gallate.

A metabolite with a Dot value > 1 is considered a key taste component, indicating its concentration in the tea infusion is perceptible [8]. In FLZY, 13 metabolites had Dot > 1 (Figure 5; Table S12), including 8 flavonol glycosides, 3 catechins, GA, and caffeine. These components may play important roles in the distinctive taste of FLZY. The eight flavonol glycosides primarily impart a velvety astringency [8]. Further analysis revealed that, except for kaempferol-3-*O*-galactoside and quercetin-3-*O*-rutinoside, which contributed key bitterness and astringency, the other six flavonol glycosides were key contributors to sweet aftertaste.

Among all metabolites, quercetin-3-*O*-rutinoside had the highest Dot value, ranging from 4036.11 to 9790.2. This compound also exhibits high Dot values in other teas, for example, 26,707.12 in Longjing tea [41]. In FLZY, the Dot value range for kaempferol-3-*O*-rutinoside was 256.70–357.59. In contrast, its Dot value in other teas is typically below 20 or 10 (e.g., <10 in Longjing tea) [41]. This suggests that kaempferol-3-*O*-rutinoside may contribute to the sweet aftertaste of FLZY.

Components with 1 > Dot > 0.1 primarily influence tea taste through interactions with other compounds [30]. In FLZY, compounds with Dot values between 0.1 and 1 were mainly umami- or sweetness-contributing amino acids and organic acids. These sub-threshold compounds may interact with key taste metabolites, contributing to FLZY’s unique taste profile.

### 3.6. Verification of Key Flavor Metabolites via Flavor Recombination and Omission Experiments

**Figure 6:**
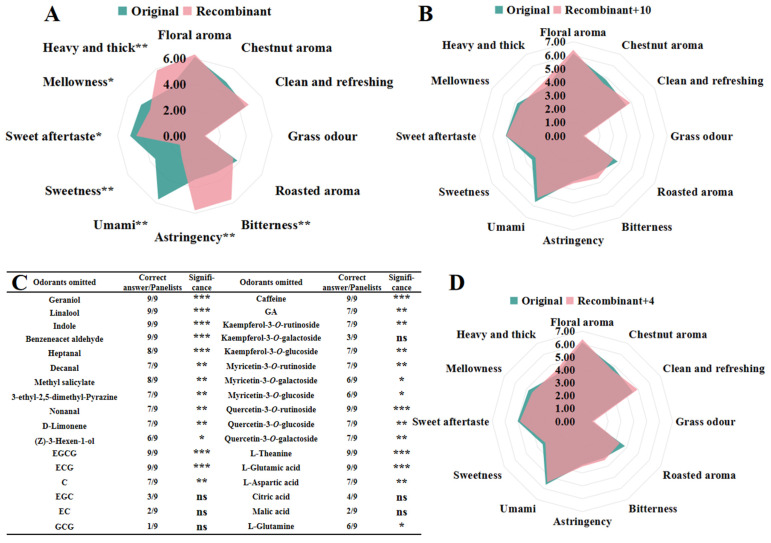
Validation of key flavor components in ‘Fuliang Zhuye 1’ (FLZY) through recombination and omission experiments. (**A**) Recombination of screened key flavor components confirms their contribution to FLZY flavor. (**B**) Recombination including components with dose-over-threshold (Dot) values below 1 reveals their substantial impact on flavor perception. (**C**) Omission of 34 flavor components identifies those essential for FLZY flavor characteristics. (**D**) Recombination with four Dot < 1 components further demonstrates their flavor contribution. * *p* < 0.05, ** *p* < 0.01, *** *p* < 0.001. GA, gallic acid; GCG, gallocatechin gallate; ECG, epigallocatechin gallate; EC, epicatechin; C, catechin; EGC, epigallocatechin; EGCG, epigallocatechin gallate.

To verify the accuracy of the 11 key aroma and 13 key taste components identified above, flavor recombination and omission experiments were conducted. Using FLZY-1 (the highest quality FLZY sample) as the control (Original), a recombinant sample (Recombinant) was prepared by adding the 11 key aroma and 13 key taste components at their respective concentrations found in the FLZY-1 infusion to pure water. QDA was used to evaluate 5 aroma and 7 taste sub-attributes. The intensities of the five aroma sub-attributes were very similar between the Recombinant and Original samples; although not perfectly overlapping, no significant differences were observed (*p* > 0.05). However, significant differences were found for all seven taste sub-attributes (*p* < 0.05) (Figure 6A). Bitterness, astringency, and “heavy and thick” mouthfeel were higher in the Recombinant, while umami, sweetness, mellowness, and sweet aftertaste were lower compared to the Original (*p* < 0.05). This suggested that the 13 key taste components selected based on Dot > 1 were insufficient to fully reconstruct the taste. Previous studies have shown that components with Dot < 1 may also contribute to the overall taste of teas like CTC black tea [42]. Therefore, we hypothesized that components in FLZY with Dot < 1 might also play key roles.

To test this, a second recombinant sample (Recombinant+10) was prepared by adding, in addition to the original 24 key components, 10 taste components with Dot values between 0.1 and 1 found in FLZY-1. These were: L-theanine, L-glutamic acid, L-aspartic acid, L-glutamine, EGC, EC, GCG, kaempferol-3-*O*-galactoside, citric acid, and malic acid. QDA evaluation revealed that both the aroma and taste profiles of Recombinant+10 and Original were very similar, with no significant differences for any sub-attribute (*p* > 0.05) (Figure 6B). The addition of these 10 components was associated with increased umami, sweetness, mellowness, and sweet aftertaste and decreased bitterness, astringency, and “heavy and thick” mouthfeel (*p* < 0.05), suggesting that some components with Dot < 1 are key flavor contributors.

Omission experiments were then conducted to investigate the contribution of individual components. In a three-alternative forced-choice test, each of the 11 aroma and 23 taste components was omitted sequentially. If the number of correct judgments by the 9 evaluators reached statistical significance, the omitted component was deemed a key flavor component, as its absence caused a significant change in flavor [28]. The results showed that 28 components reached significance (*p* < 0.05): all 11 aroma components and 17 taste components (Figure 6C). This indicated that the initially selected 11 aroma and 13 taste components (Dot > 1) were indeed key. Among the 10 components with Dot < 1 tested, 4 showed significant differences when omitted: L-theanine and L-glutamic acid (*p* < 0.001), L-aspartic acid and L-glutamine (*p* < 0.01). The other 6 components (EGC, EC, GCG, kaempferol-3-*O*-galactoside, citric acid, malic acid) showed no significant difference (*p* > 0.05), indicating they are not key taste components.

Consequently, a third recombinant sample (Recombinant+4) was prepared by adding only the 4 newly suggested key components (L-theanine, L-glutamic acid, L-aspartic acid, L-glutamine) along with the original 24 key components to pure water. QDA showed no significant differences between Recombinant+4 and Original for any aroma or taste sub-attribute (*p* > 0.05) (Figure 6D), further suggesting the key roles of these four amino acids. Their addition was associated with increased umami, sweetness, mellowness, and sweet aftertaste in the recombinant by 171.1%, 145.2%, 11.9%, and 6.6%, respectively, and decreased bitterness, astringency, and “heavy and thick” mouthfeel by 39.1%, 39.3%, and 25.2%, respectively.

In conclusion, the three recombination experiments and the omission experiments collectively identified 28 key flavor components for FLZY. Among these, the four amino acids with Dot < 1—L-theanine, L-glutamic acid, L-aspartic acid, and L-glutamine—appeared to play important roles in enhancing umami, sweetness, mellowness, and sweet aftertaste while reducing bitterness, astringency, and “heavy and thick” mouthfeel in FLZY.

It should be noted that the nominal concentrations were based on the levels originally detected in the FLZY-1 tea infusions. A limitation of this study is the lack of independent analytical validation for the final concentrations in the recombination buffers. Additionally, we did not statistically evaluate potential synergistic or antagonistic interactions among metabolites in our recombination and omission experiments. Therefore, whether the observed flavor contributions are additive or involve non-linear interactions remains unknown, and future studies using factorial designs or mixture models are required to address this question.

### 3.7. Metabolic Pathways of Key Flavor Metabolites in FLZY and the Influence of Spring Seasonal Changes

**Figure 7:**
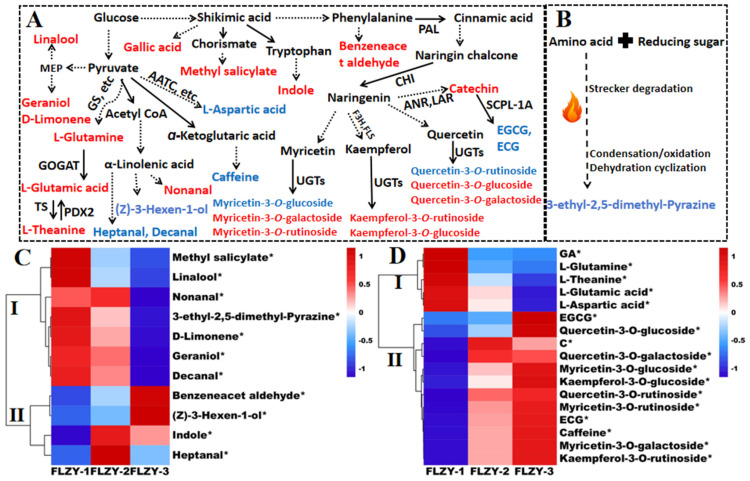
Proposed metabolic pathways and seasonal effects on key flavor metabolites in ‘Fuliang Zhuye 1’ (FLZY). (**A**) Biosynthetic pathways of key flavor metabolites in tea plants. (**B**) Biochemical synthesis pathways of key flavor metabolites during tea processing. The red font indicates that the metabolite has a high content in FLZY, while the blue font indicates a high content in ‘Fuding Dabaicha 1’ (FDDB). The solid line represents a single-step biosynthesis, while the dotted line represents a multi-step biosynthesis. (**C**) Effects of spring seasonal changes on key aroma metabolites in FLZY. (**D**) Effects of spring seasonal changes on key taste metabolites in FLZY. * *p* < 0.05. Red indicates high content and blue indicates low content. I and II represent being divided into two categories. GOGAT, glutamate synthase; TS, theanine synthetase; PDX2, pyridoxine biosynthesis 2; GS, glutamine synthetase; PAL, phenylalanine ammonia-lyase; AATC, aspartate aminotransferase cytoplasmic; CHI, chalcone isomerase; LAR, leucoanthocyanidin reductase; ANR, anthocyanidin reductase; SCPL-1A, serine carboxypeptidase-like clade 1A; F3H, flavonoid 3-hydroxylase; FLS, flavonol synthase; UGTs, UDP-glucose flavonoid-3-*O*-glucosyl transferases; MEP, methylerythritol phosphate; GA, gallic acid; ECG, epigallocatechin gallate; C, catechin; EGCG, epigallocatechin gallate.

Figure 7A illustrates the metabolic pathways of 28 key flavor metabolites in tea plants. Catechins and flavonol glycosides are primarily synthesized through the flavonoid pathway [31]. In FLZY, flavonol glycosides and non-ester catechins showed high accumulation, whereas ester catechins showed low accumulation. This may be attributed to the relatively high expression of UDP-glucose flavonoid-3-*O*-glucosyl transferases (UGTs) and anthocyanidin reductase (ANR), coupled with the relatively low expression of serine carboxypeptidase-like clade 1A (SCPL-1A). The contents of L-theanine, L-glutamic acid, and L-glutamine were elevated, which speculatively may be related to high expression of glutamate synthase (GOGAT), theanine synthetase (TS), and glutamine synthetase (GS). Linalool, geraniol, and D-limonene were present in relatively high amounts, whereas (Z)-3-hexen-1-ol, heptanal, and decanal were relatively low. This pattern might be associated with a relatively active methylerythritol phosphate (MEP) pathway and a comparatively weak *α*-linolenic acid metabolism pathway. Figure 7B shows 3-ethyl-2,5-dimethyl-pyrazine, which is generated during the heat treatment of green tea processing. It is formed via the Strecker degradation of amino acids and reducing sugars under heating conditions [37]. Although FLZY exhibited relatively high amino acid content, its glucose content was low, which may help explain its low pyrazine content. Overall, core pathways—including phenylalanine metabolism, flavonoid biosynthesis, amino acid metabolism, *α*-linolenic acid metabolism, and MEP metabolism in tea plants, as well as Strecker degradation during tea processing—may have played an important role in shaping the unique flavor profile of FLZY.

### 3.8. Influence of Spring Seasonal Changes on Key Flavor Metabolites in FLZY

In the QDA, spring seasonal changes were found to alter the flavor of FLZY. Furthermore, the influence of seasonal variation on key flavor metabolites was analyzed. The effect of spring seasonal changes on 11 key aroma components could be classified into two categories (Figure 7C). The first category included seven key aroma components that showed a decreasing trend from early to late spring (*p* < 0.05): geraniol, linalool, and D-limonene, which contribute to floral aroma; 3-ethyl-2,5-dimethyl-pyrazine, which contributes to roasted aroma; and methyl salicylate and nonanal, which contribute to clean and refreshing notes. The second category comprised four key aroma components that exhibited an increasing trend (*p* < 0.05), including (Z)-3-hexen-1-ol, which contributes to grassy odor; heptanal, associated with chestnut aroma; and indole and benzaldehyde, which contribute to floral aroma. Previous studies have confirmed that tea aroma is highest in autumn, followed by spring and summer, and weakens from spring to summer [43], which is consistent with the findings of this study. In spring, many floral aroma metabolites in FLZY decreased seasonally, which may reduce its overall aromatic quality.

The influence of spring seasonal changes on 17 key taste components could also be divided into two categories (Figure 7D). The first category included five key taste components with a decreasing trend (*p* < 0.05): L-theanine, L-glutamine, L-glutamic acid, and L-aspartic acid, which contribute to umami and sweetness. The second category consisted of 12 taste components with an increasing trend (*p* < 0.05), primarily including flavonol glycosides that contribute to a sweet aftertaste, as well as catechins and caffeine that contribute to astringency, bitterness, and heavy and thick characteristics. Previous studies have reported that from early to late spring, carbon metabolism and flavonoid biosynthesis gradually increase, while nitrogen metabolism and amino acid synthesis weaken. This leads to an upward trend in flavonoid content and a downward trend in amino acids in tea plants [24]. Additionally, recent research has shown that the high expression of *CsTHS1* and *CsGDH2.1* downregulates theanine content in tea plants during late spring [44], which is consistent with the results of this study.

In summary, metabolites that contribute key superior flavor attributes (e.g., floral aroma and umami) decrease seasonally in spring, while those contributing to grassy odor, astringency, and bitterness increase seasonally. These seasonal changes may contribute to a decline in the flavor quality of FLZY.

## 4. Conclusions

In conclusion, the influence of tea plant cultivars on metabolites in Fuliang tea was greater than that of seasonal changes. Compared with FDDB, the Fuliang tea produced by FLZY better reflected its traditional flavor profile, characterized by floral aroma and sweet aftertaste. Multimodal flavoromics identified 28 key flavor components contributing to the unique flavor of FLZY. These components may be synthesized through several core metabolic pathways, including phenylalanine metabolism, flavonoid biosynthesis, amino acid metabolism, *α*-linolenic acid metabolism, and the MEP pathway, as well as Strecker degradation during processing. Flavor recombination and omission experiments suggested that four components with Dot values less than 1—L-theanine, L-glutamine, L-glutamic acid, and L-aspartic acid—were key taste components. They enhanced the umami, sweetness, mellowness, and sweet aftertaste of FLZY while reducing its bitterness, astringency, and heavy and thick mouthfeel. Additionally, kaempferol-3-*O*-rutinoside accumulated to notably high levels in FLZY (11.75 times that in FDDB), suggesting a possible role in the enhanced sweet aftertaste. Compared with early spring, the quality of FLZY declined in late spring, which may be due to the seasonal decrease in metabolites contributing to floral aroma and umami, combined with a seasonal increase in metabolites contributing to grassy odor, astringency, and bitterness. Nevertheless, the findings of this study should be considered in light of certain limitations. The absence of environmental replication and independent validation cohorts, due to the single-origin sampling design, means that this work provides an exploratory rather than confirmatory assessment of cultivar-season interactions. Future research with multi-site, multi-year sampling is warranted to validate the metabolic basis of Fuliang tea flavor proposed here.

## Data Availability

The original contributions presented in this study are included in the article. Further inquiries can be directed to the corresponding authors.

## Supplementary Materials

The following supporting information can be downloaded at: https://www.mdpi.com/article/10.3390/plants15111635/s1, Figure S1: PLS-DA model for volatile metabolites of all samples; Figure S2: PLS-DA model for no-volatile metabolites of all samples; Table S1: Taste an aroma descriptors and their definitions; Table S2: Sensory evaluation results of the aroma of the sample. Table S3: Sensory evaluation results of the taste of the sample. Table S4: Identification results of volatile metabolites in all samples (µg/kg); Table S5: Screen for differential volatile metabolites across all samples; Table S6: The correlation between important aroma metabolites and aroma properties; Table S7: GC-O identified the aroma components of FLZY; Table S8: Non-volatile metabolites identified in all samples; Table S9: Selected differential non-volatile metabolites; Table S10: The correlation between important taste metabolites and taste attributes; Table S11: Absolute quantification of chemical composition (mg/g); Table S12: Dot value of chemical composition.
