# Supplementary material for: Analysis of Unique Flavor Metabolites and Seasonal Variations of the Special Tea Plant Cultivar of Fuliang Tea, a Geographical Indication Product
Source: Plants (Basel). 2026 May 26;15(11):1635. doi: 10.3390/plants15111635 (PMC13259503; doi:10.3390/plants15111635)
Supplement: Supplementary file 1 [file plants-15-01635-s001.zip › Supporting figures.pdf]

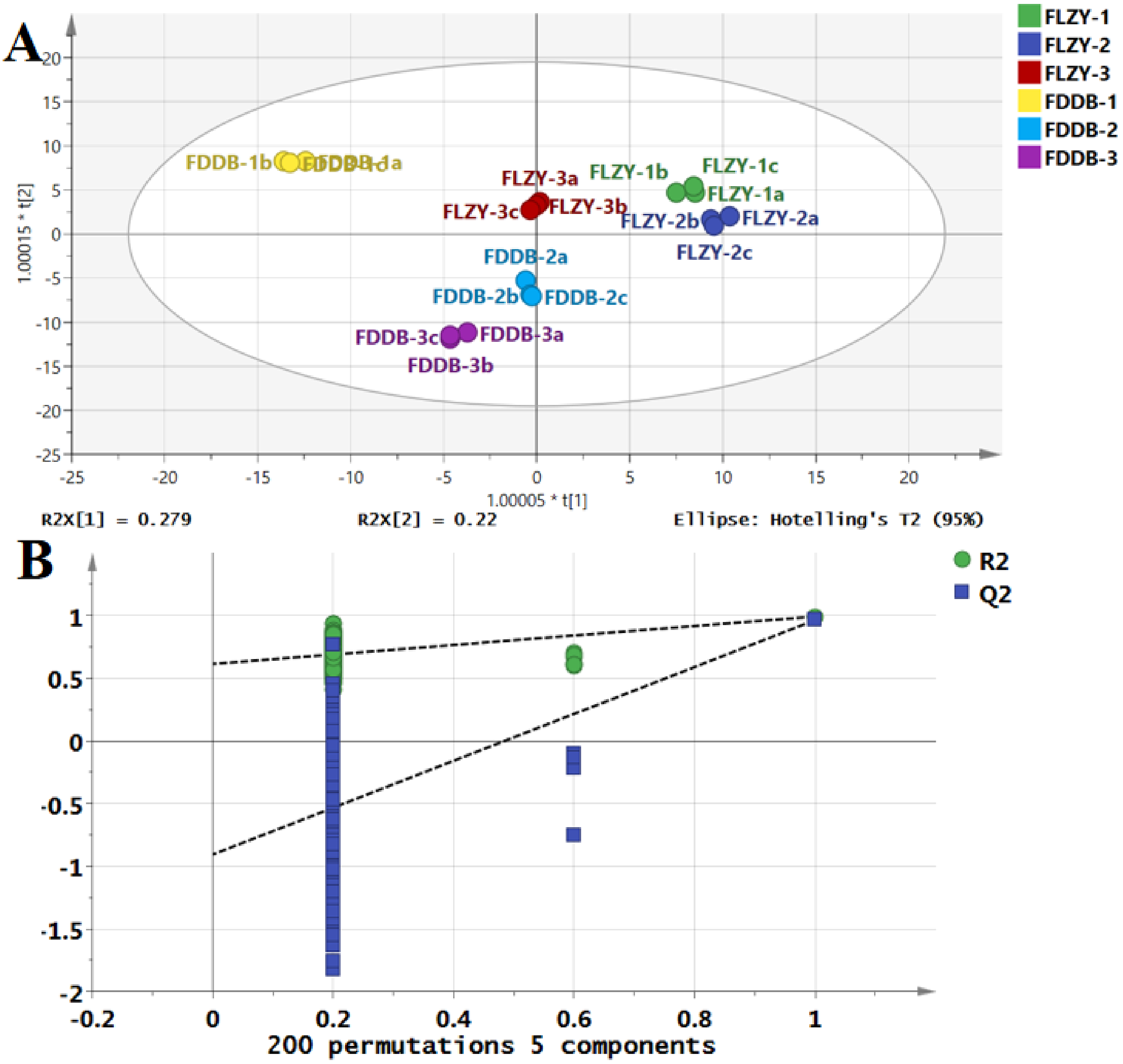

Fig.S1. PLS-DA model for volatile metabolites of all samples. (A) PLS-DA score plot. Seven-fold cross-validation was used. The model exhibited high explanatory and predictive power ($R^2X = 0.901$, $R^2Y = 0.992$, $Q^2 = 0.949$). (B) Permutation test results (200 permutations). The intercepts for R^2 and Q^2 were 0.614 and -0.907, respectively. The negative Q^2 intercept (-0.907) confirmed that the original model was not overfitted, demonstrating its statistical robustness.

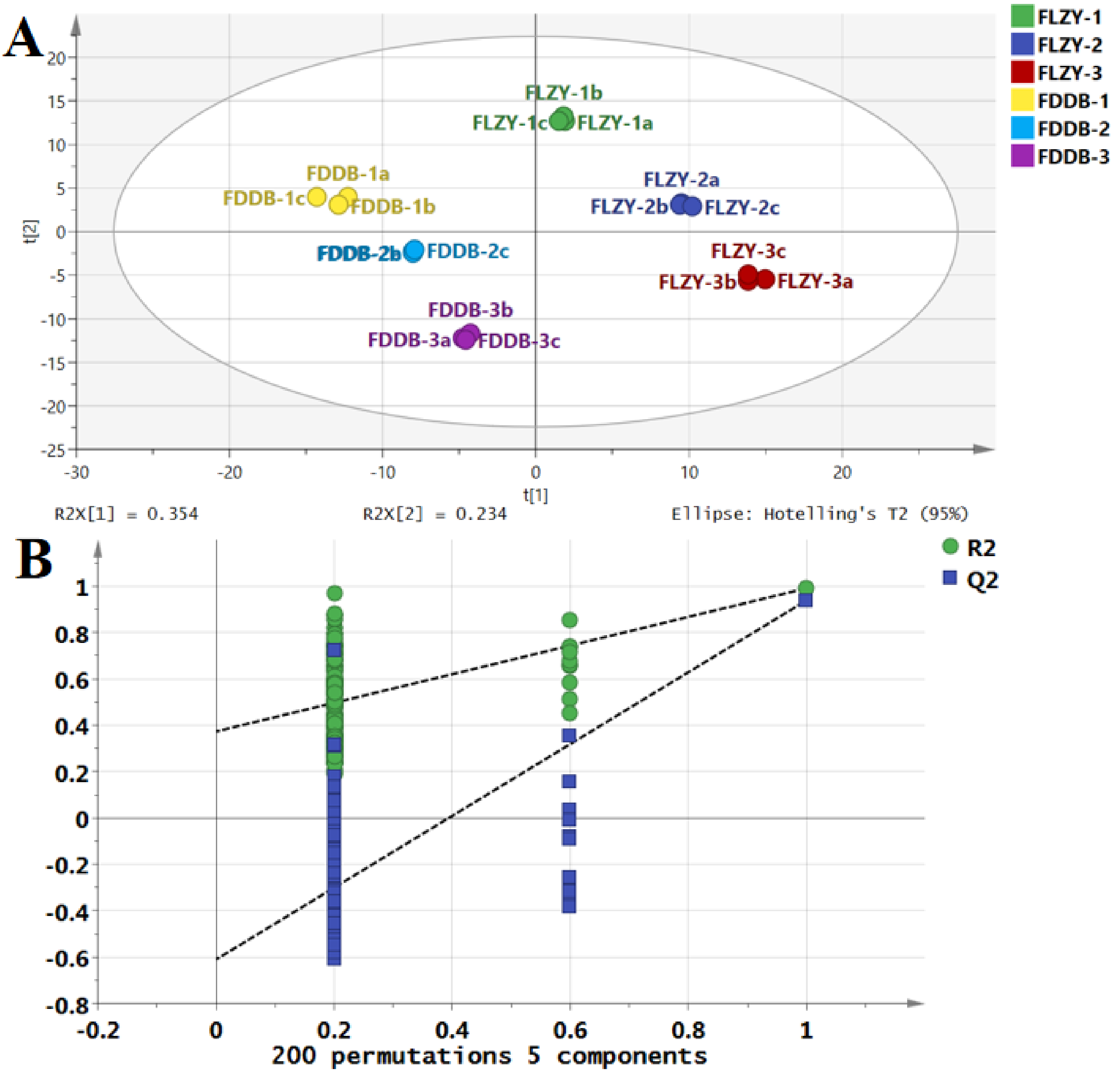

Fig.S2. PLS-DA model for no-volatile metabolites of all samples. (A) PLS-DA score plot. Seven-fold cross-validation was used. The model exhibited high explanatory and predictive power ($R^2X = 0.811$, $R^2Y = 0.992$, $Q^2 = 0.924$). (B) Permutation test results (200 permutations). The intercepts for R^2 and Q^2 were 0.373 and -0.61, respectively. The negative Q^2 intercept (-0.610) confirmed that the original model was not overfitted, demonstrating its statistical robustness.

.
